# Residue Behavior of Methoxyfenozide and Pymetrozine in Chinese Cabbage and Their Health Risk Assessment

**DOI:** 10.3390/foods11192995

**Published:** 2022-09-26

**Authors:** Wenting Wang, Yu-Jin Cho, Jong-Wook Song, Yeong-Jin Kim, Jong-Su Seo, Jong-Hwan Kim

**Affiliations:** Environmental Safety-Assessment Center, Korea Institute of Toxicology (KIT), Jinju 52834, Korea

**Keywords:** pesticides, Chinese cabbage, dissipation pattern, temperature effect, risk quotient

## Abstract

Methoxyfenozide and pymetrozine are used for pest control in the cultivation of Chinese cabbage. This has raised concerns in recent years due to health risks. Therefore, this study aimed to determine the residual concentrations of pesticides in the target crop and associated health risks. The dynamics and influence of environmental factors on the dissipation of methoxyfenozide and pymetrozine residues in Chinese cabbage were investigated. Analyses were performed using a modified QuEchERS (Quick, Easy, Cheap, Effective, Rugged, and Safe) and an optimized high-performance liquid chromatography-tandem mass spectrometry (HPLC-MS/MS). The observed half-lives of methoxyfenozide and pymetrozine in cabbage samples ranged between two sampling seasons: in May–June, half-lives of methoxyfenozide and pymetrozine were 1.20 days and 1.89 days, respectively; during October–November, half-lives of methoxyfenozide and pymetrozine were 11.8 and 2.80 days, respectively. Meanwhile, a negative Spearman correlation was found between the residual concentrations and temperature (*p* < 0.01). This indicates that higher temperatures resulted in higher dissipation rates for methoxyfenozide and pymetrozine, suggesting that these pesticides degraded faster at higher temperatures. Additionally, higher pesticide residues in Chinese cabbage during low-temperature seasons resulted in higher risk quotients (RQ) (RQ > 1) for both analyzed compounds, which suggests that the effect of temperature on pesticide degradation needs to be considered as an essential factor while setting up the maximum residue limits (MRL).

## 1. Introduction

Chinese cabbage is cultivated and consumed globally and is particularly popular in Asia [1,2]. For example, it is the main ingredient of the traditional Korean dish called “Kimchi,” consumed daily by Koreans and has been growing in popularity in the West [3]. It has been reported that, for Koreans, the average daily consumption of kimchi is 42.0–63.9 g/d from Korea Health Industry Development Institute. Consumption of high amounts of Chinese cabbage is raising health concerns due to the pesticides used in the field. Lepidopteran larvae are one of the main pests of Chinese cabbage. Methoxyfenozide insecticide is highly effective in inducing premature, incomplete ecdysis and death [4]. Other hazardous pests are cabbage aphids, and pymetrozine is widely used against them [5]. According to the United States Environmental Protection Agency (U.S. EPA), pymetrozine is a “likely” human carcinogen, and there are insufficient data to eliminate the need for a quantitative risk assessment of the substance. While the observed effect level in 90-day exposures were the highest test concentration (1369 mg/kg/day) observed in rat [6].

Therefore, some studies have investigated the dissipation behaviors of methoxyfenozide and pymetrozine. Previous studies have used a modified QuEChERS method with liquid chromatography-tandem mass spectrometry (LC-MS/MS) to quantify methoxyfenozide residues in food [2,7,8,9]. However, inconsistent pretreatment methods were reported for pymetrozine, combining QuEChERS and solid phase extraction with cartridges, and liquid-liquid extraction methods have been applied for pymetrozine analysis [10,11,12,13]. Thus, it is necessary to establish an optimized pretreatment method to analyze the pesticide residues in Chinese cabbage.

A variability in pesticide dissipation half-lives in crops has been reported, affected by various factors [14,15,16], mainly environmental factors including temperature, pH, moisture, rainfall, and photodegradation, etc. [17,18,19,20]. In particular, the effects of temperature have been confirmed for pesticides such as isoproturon, metribuzin, terbuthylazine, and chlorpyrifos in fruits and vegetables [14,21,22]. However, no related studies have investigated the effect of environmental conditions on the dissipation of methoxyfenozide and pymetrozine in fruits or vegetables.

The present study aimed to determine the dissipation patterns of methoxyfenozide and pymetrozine in Chinese cabbage. The effects of different environmental conditions on cabbage were also evaluated during different sampling periods. Additionally, a dietary risk assessment was conducted for both compounds. The results can serve as a reference for farmers regarding the proper application of methoxyfenozide and pymetrozine.

## 2. Materials and Methods

### 2.1. Reagents and Materials

Analytical grade methoxyfenozide (99.6% purity) and pymetrozine (99.5%) were purchased from HPC Standard GmbH (Cunnersdorf, Germany). Methanol, water, and acetonitrile were purchased from Burdick & Jackson (Muskegon, MI, USA). Reagent grade formic acid (>98%) and sodium tetraborate decahydrate were obtained from Sigma-Aldrich (Burlington, MA, USA). The QuEChERS extraction kit was obtained from Phenomenex (Torrance, CA, USA).

### 2.2. Crop Field Trials

For crop field trials, Chinese cabbage plants were grown in three locations in the Republic of Korea: Jinju (JJ-19-6; 35°12′ N, 128°18′ E), Yeongdeok (YD-19-8; 36°24′ N, 129°18′ E), and Gimhae (GH-19-9; 35°15′ N, 128°59′ E) in accordance with Organisation for Economic Co-operation and Development guidelines for the testing of chemicals (TG No. 509) [23] (Figure S1). Each trial comprised triplicates of treated and untreated (control) plots of areas 10.2–25.0 m^2^, cultivated with similar agriculture practices. Prior to commencement, all trial sites were assessed to ensure no history of methoxyfenozide or pymetrozine application. In addition, we made sure that the target compounds used in the trials would not contaminate the surrounding area. Buffer zones of 1.0–1.5 m were created between the treated and untreated plots to avoid pesticide drift.

Commercial products, a suspension concentrate formulation containing 21% of the active ingredient (a.i.) methoxyfenozide (Runner, Farm Hannong Co., Ltd., Seoul, Korea), and a water dispersible granule formulation containing 50% of a.i. pymetrozine (Minecto star, Syngenta Co., Ltd., Seoul, Korea) were diluted with water and sprayed three times at intervals of approximately 7 days on separate plots, following the guidelines of the Korea Crop Protection Association (2019). Pesticide solutions were not applied during strong winds or rainfall events to prevent spreading and ensure effective application. Test periods, soil types, dilution rates, spray intervals, and application rates were collected on time (Table 1).

### 2.3. Crop Sampling

During the sample collection, crops from the plot edges and row ends were excluded. In addition, to minimize the possibility of cross-contamination, samples from the control plots were collected before the treated plots. Trial crops were harvested 2–3 h and 1, 3, 5, 7, and 14 days after the last pesticide application. The pre-harvest intervals (PHI) of 7 and 14 days were set for methoxyfenozide and pymetrozine, respectively, in Chinese cabbage. Additionally, different sampling periods were used to investigate the effect of environmental conditions on pesticide residue content. The environmental conditions of the trial period, including temperature, humidity, rainfall, and wind velocity, are listed in Table S1.

From each plot, 4–6 cabbages were collected, weighing over 5 kg in total. After removing the outer leaves, any obvious decomposed/withered leaves, and adhering soil, the cabbages were placed in double-labeled bags, then transported to the test facility using a temperature-controlled container (4–9 °C). All samples were weighed, chopped, and homogenized by blender (DA333-G, Daesung Artlon, Gyeonggi-do, Korea). Subsequently, 10 g of each sample was weighed using a balance scale and stored at a temperature of −20 °C.

### 2.4. Sample Preparation and Analysis

Three replicate samples of each sampling period were pretreated by a modified QuEChERS method [24] and analyzed as follows: 10 and 20 mL of acetonitrile were added separately in each 10 g of crop sample for methoxyfenozide and pymetrozine pretreatment, respectively, by shaking for 10 s. Subsequently, 4 g of magnesium sulfate, 1 g of sodium chloride, 1 g of sodium citrate tribasic dihydrate, and 0.5 g of sodium citrate dibasic sesquihydrate were added for extraction. For samples treated with pymetrozine, 3 mL of 0.5 M borate buffer was added to adjust the pH and improve the recovery of the pesticide. The substance was then mixed for 3 min using an automated shaker at 1000 rpm. Thereafter, the samples were centrifuged for 5 min at 4500 rpm, at 4 °C. The resulting supernatants were then separated using a 0.2-µm syringe filter, and the filtrates thus obtained were diluted 10- and 5-fold with acetonitrile and analyzed using HPLC-MS/MS (Agilent 1260/6460 QQQMSD system version B.06.00, Santa Clara, CA, USA) in electrospray ionization mode.

### 2.5. Analytical Method Validation

The analytical method was validated using specificity, linearity, and recovery tests to evaluate the accuracy (recovery rate, %) and precision (relative standard deviation, RSD, %) of methoxyfenozide and pymetrozine. The limit of quantitation (LOQ) was greater than the signal-to-noise ratio of 10:1. Ten grams of untreated samples were fortified at different levels, namely, LOQ (0.01 mg/kg), 10 (0.1 mg/kg), and 50 times (0.5 mg/kg) the LOQ level, five replicates were performed separately for each pesticide. Six concentrations of calibration curve ranging from 0.0005 to 0.1 mg/L (0.0005, 0.002, 0.005, 0.01, 0.05, and 0.1 mg/L) were used to assess the linearity. The recovery rates were determined by comparing the peak areas of the fortified samples using a matrix-matched calibration curve.

### 2.6. Analytical Quality Control and Sample Storage Stability

To verify the accuracy of the HPLC-MS/MS analysis of the samples, analytical quality control (AQC) samples (0.05 mg/kg) were prepared and placed at the beginning and end of the test runs or between the runs. The details of the HPLC-MS/MS conditions, including information on the mobile phase, column, and multiple reaction monitoring (MRM), are presented in Table S2.

A storage stability test of residue was carried out to evaluate the stability of residues in Chinese cabbage. Thus, the test pesticide solutions were added to the control Chinese cabbage sample to a final concentration of 0.5 mg/kg as a fortification experiment and then stored under the same conditions (−20 °C) and periods as the field samples were harvested. They were then pretreated in the same manner as the field trial samples.

### 2.7. Calculation of Half-Lives

The dissipation process of the target compounds in Chinese cabbage was evaluated against time, using the first-order kinetic Equation [25]. Therefore, the dissipation dynamics and half-lives were calculated as follows:

C_t_ = C_0_e^−kt^, (1)

t_1/2_ = ln(2)/k, (2)

where C_t_ (mg/kg) represents the concentration of the residual pesticides at time t (days), C_0_ (mg/kg) is the initial concentration of the residual pesticide at time t = 0, and k (day^−1^) represents the degradation coefficient.

### 2.8. Dietary Risk Assessment

The risks from consumption of the Chinese cabbage contaminated with residues of pesticides were assessed through quantification of the estimated daily intake (EDI). We used the following equation provided by the US EPA [26] to calculate the EDI of target compounds by the population of the Republic of Korea:

EDI = C × R/BW,(3)

where C (mg/kg) is the median concentration of methoxyfenozide (PHI = 7 days) and pymetrozine (PHI = 14 days) found in Chinese cabbage, R (g/d) is the consumption rate of the cabbage, BW (kg) is the average body weight.

Finally, we evaluated the chronic risk posed by pesticide residues in the Chinese cabbage through risk quotient (RQ) by comparing the EDI with acceptable daily intake (ADI) using the following equation:

RQ = EDI/ADI,(4)

### 2.9. Statistical Analysis

Statistical analyses were performed using SigmaPlot version 12.0 for Windows software (Systat Software, Inc., San Jose, CA, USA). The half-life calculation parameters were obtained by conducting nonlinear regression. Correlation coefficients were determined by performing Spearman correlation analyses. A probability (*p*) value < 0.05 was considered to indicate statistical significance.

## 3. Results

### 3.1. Analytical Method Validation

To optimize the MS/MS conditions, 500 ng/mL of each standard solution dissolved in acetonitrile was injected into the detector. Total ion chromatography (TIC) in positive mode was obtained by scanning at 50–500 *m*/*z*. The most abundant ions were selected as the precursor ions. Quantifier and qualifier ions were the highest and second-highest intensities selected by changing various parameters, such as fragment voltage and collision energy (Table 2).

The limit of detection (LOD) and LOQ were 0.005 and 0.01 mg/kg for both pesticides, respectively. HPLC-MS/MS revealed no interference peaks in control samples from all trial sites, indicating the absence of interference during sample analysis. Matrix-matched calibrations for methoxyfenozide and pymetrozine were used to compensate for the matrix effects, resulting in satisfactory linearity (R^2^ > 0.998).

**Figure 1:**
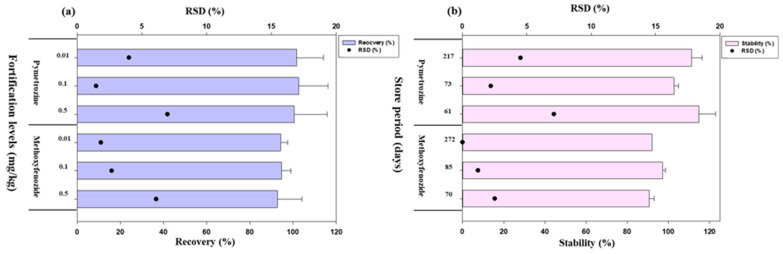
(**a**) Recoveries (%) and RSD (%) under different fortification levels (mg/kg) and (**b**) storage stabilities (%) and RSD (%) for different store period (days) at below −20 °C observed for methoxyfenozide and pymetrozine in Chinese cabbage.

Acetonitrile and acetone have generally been used as extraction solvents for agricultural products; thus, it has been reported that these solvents have high extraction efficiency in the process of extracting methoxyfenozide from agricultural products [27,28]. However, it is reported that pymetrozine is a highly polar pesticide (log Pow: −0.18, water solubility: 0.29 g/L), and it is necessary to adjust the pH by adding a weakly basic buffer solution to suppress ionization to achieve a high extraction efficiency [29,30]. Therefore, 0.5 M borate buffer solution was applied as extraction solvent to extract the pymetrozine. As shown in Figure 1a, mean recoveries for methoxyfenozide ranged from 80.0 to 104.0%, with a relative standard deviation (RSD) less than 6.1%, and ranged from 83.4–120.0% with an RSD less than 6.9% for pymetrozine. These results gave satisfactory recoveries between 70% and 120% with an RSD < 20%, according to the European Commission Directorate-General for Health and Food Safety guidelines. Additionally, the chromatograms for methoxyfenozide and pymetrozine analyzed by HPLC-MS/MS were shown in Figure S2.

### 3.2. Analytical Quality Control and Storage Stability of Samples

To ensure the quality and comparability of the analytical results, AQC was performed [31]. The AQC recovery results were in the range of 96.0–106.0% for methoxyfenozide and 100.0–102.0% for pymetrozine.

The storage stability test of residue was carried out to evaluate the stability of residues in Chinese cabbage because the degradation, decay, and dissipation of residues could occur even under freezing conditions of below −20 °C [32]. As shown in Figure 1b, the mean stabilities (*n* = 3) of methoxyfenozide ranged between 90.7–97.3%, with RSDs lower than 2.5%, and between 102.7–111.3%, with RSDs lower than 7.1% for pymetrozine.

A storage stability test showed almost no degradation and demonstrated the stability of each pesticide. In accordance with the Joint FAO/WHO Meeting on Pesticide Residues Training Manual, the analytes were defined as stable if the degradation rate was less than 30%.

### 3.3. Dissipation Patterns

**Figure 2:**
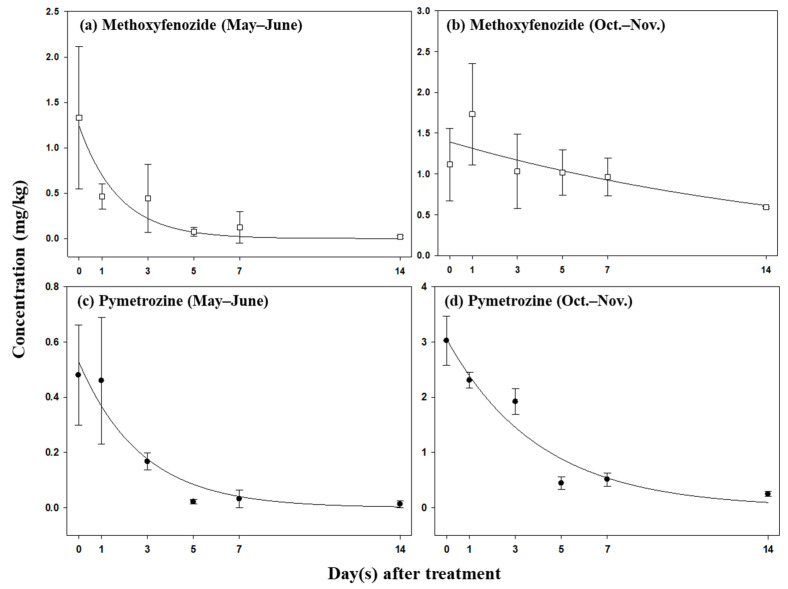
Residue decline trends of (**a**,**b**) methoxyfenozide and (**c**,**d**) pymetrozine during 0–14 days in the different sampling periods.

The dissipation patterns of methoxyfenozide and pymetrozine in Chinese cabbage collected during the different sampling periods are presented in Figure 2.

The initial concentrations of methoxyfenozide and pymetrozine were detected at 1.33 ± 0.79 and 0.48 ± 0.18 mg/kg for crops collected during May–June and at 1.12 ± 0.45 and 3.02 ± 0.44 mg/kg for those collected during October–November, respectively. The residual amounts of methoxyfenozide and pymetrozine on day 14 were 0.02 ± 0.01 and 0.02 ± 0.01 mg/kg for May–June, and 0.59 ± 0.01 and 0.25 ± 0.05 mg/kg for October–November, respectively. The initial detected concentrations collected for methoxyfenozide in both periods were below the MRL for methoxyfenozide (2.0 mg/kg) for the Ministry of Food and Drug Safety, Republic of Korea, and the residuals were also within the MRL reported by Codex Alimentarius International Food Standards for cabbage (7 mg/kg). Meanwhile, the residual levels from 3 days (0.17 ± 0.03 mg/kg) to 14 days (0.02 ± 0.01 mg/kg) for pymetrozine collected during May and June were below the MRL (0.2 mg/kg) for South Korea. However, no MRL for pymetrozine in the Chinese cabbage has been set in the Codex Standards thus far. The residual concentrations during October and November were higher than the MRL regulated for pymetrozine, even though we used the recommended application doses. These results suggested that low temperatures may slow down the pesticide dissipation, thus affecting the residual concentrations in crops. It is also reported that the six pesticides (dimethomorph, imidaclothiz, lufenuron, methoxyfenozide, pyridaben, and spinetoram) exhibited different residual concentrations (approximately more than 2–3 times) in park choi at different field trials where the air temperature was relatively different (low, 4–9 °C; high, 12–25 °C) [2].

Therefore, to investigate the effects of environmental factors on the pesticide dissipation rate in cabbages, four factors, including temperature, humidity, rainfall, and wind velocity, were measured during the test periods (Table S1). However, no statistically significant differences were found in humidity, rainfall, and wind velocity.

**Figure 3:**
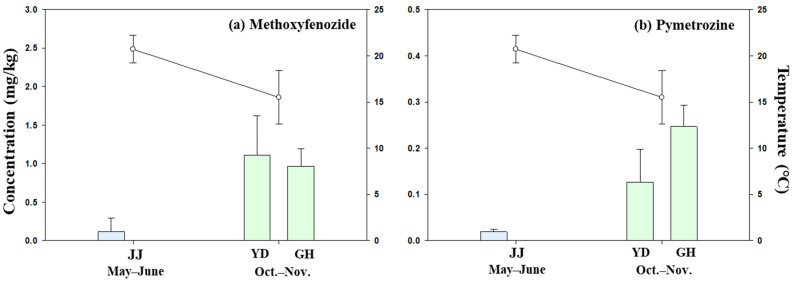
Effects of temperature on residual concentration of (**a**) methoxyfenozide, and (**b**) pymetrozine in Chinese cabbages.

As shown in Figure 3, Chinese cabbage samples collected during each season showed negative Spearman correlations between the concentrations of pesticides and temperature (*p* < 0.01). This indicates that higher temperatures resulted in higher dissipation rates for methoxyfenozide and pymetrozine. It suggested that both chemicals degraded faster at higher temperatures as reported elsewhere for other pesticides in both field- and laboratory-scale experiments [21,22,33].

Thus, the half-lives of methoxyfenozide and pymetrozine in Chinese cabbage collected from different sampling periods were calculated using Equations (1) and (2), as shown in Table 3. The half-lives ranged from 1.20 to 11.8 days for methoxyfenozide and from 1.89 to 2.80 days for pymetrozine during the different sampling periods.

Until now, only a few studies have reported the half-lives of both pesticides in different crops. The half-lives of methoxyfenozide have previously been reported to be 2.5–3.5 days in cauliflower and 1.2 days in tea [7], which are lower than those observed in our study. Another study conducted on pak choi reported similar half-lives of methoxyfenozide (3.9–8.6 days) under open field and greenhouse conditions [2], suggesting that environmental factors, particularly temperature, influence the degradation of methoxyfenozide as mentioned above. The half-lives of pymetrozine in Chinese cabbage crops observed in this study were lower than those estimated in Egyptian strawberries (3.2 days), Chinese cauliflower (<4 days), and Chinese kale (3.0–4.1 days) for pymetrozine [5,34,35], indicating the differences among half-lives of these crops.

### 3.4. Dietary Risk Assessment

To evaluate the potential health risks to humans posed by Chinese cabbage consumption, median concentrations of methoxyfenozide and pymetrozine at PHI = 7 days and 14 days, respectively, were used. The estimated EDI values of the target pesticides were compared with the ADI values provided by the National Institute of Agricultural Sciences. The ADIs for methoxyfenozide and pymetrozine were 0.11 and 0.03 mg/kg body weight/day. The Korea Health Industry Development Institute and the National Survey of Exposure Factors for Korean Adults and Children provided data on the average body weight and cabbage consumption in South Korea, as shown in Table S3.

The EDIs (in mg/kg body weight/day) of the target pesticides were estimated. In terms of sex, the EDI values were higher in males, 0.020–0.732 for methoxyfenozide and 0.017–0.192 for pymetrozine. Conversely, lower EDI values for methoxyfenozide (0.017–0.605) and pymetrozine (0.014–0.158) were observed in females. This can be explained by the fact that males consume cabbage daily at a higher rate (63.9 g/d) than females (42.0 g/d) (Table S3).

**Figure 4:**
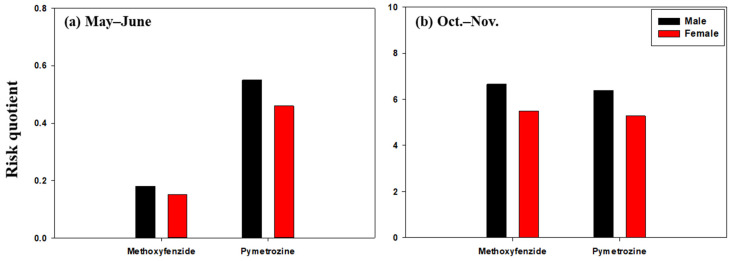
Risk quotient for methoxyfenozide and pymetrozine in cabbage collected in (**a**) May–June and (**b**) October–November.

In addition, the RQ, which indicates the chronic risks posed by contaminants, was calculated from the EDI and reported ADI values for the analyzed pesticides, as shown in Figure 4. An RQ value higher than 1 indicates that the risk of pesticides to humans is unacceptable. In contrast, an RQ value of less than 1 represents a minimal risk to humans [36]. The estimated RQs of the two target compounds in Chinese cabbage were observed at different magnitudes during different sampling periods. During May and June, the RQs for methoxyfenozide ranged from 0.15–0.18 and for pymetrozine ranged from 0.46–0.55, respectively, whereas the RQ values of trial crops collected during October and November (methoxyfenozide: 5.50–6.66; pymetrozine: 5.28–6.39) were approximately 10–50 times higher than those of May and June. This suggested that higher pesticide residues during the cold seasons resulted in higher RQ values for both compounds. Thus, setting PHI to longer than 7 days for methoxyfenozide and 14 days for pymetrozine during low-temperature seasons could result in lower health risks.

## 4. Conclusions

The dissipation dynamics of pesticides in Chinese cabbage crop trials collected were evaluated. In crops collected in May and June, the residual concentrations of methoxyfenozide ranged from 0.02 to 1.33 mg/kg and from 0.02 to 0.48 mg/kg for pymetrozine. Higher residual concentrations were observed during October and November (methoxyfenozide: 0.59–1.12 mg/kg; pymetrozine: 0.25–3.02 mg/kg). The Chinese cabbage samples collected from each trial showed negative Spearman correlations between the concentrations of pesticides and temperature (*p* < 0.01), which suggested a temperature effect on the half-lives of methoxyfenozide (1.20–11.8 days) and pymetrozine (1.89–2.80 days). Moreover, the dietary risk assessment was also estimated based on Chinese cabbage consumption. Remarkably, the RQ values estimated for Chinese cabbage collected during October and November were much higher than those collected during May and June, even though the residual concentrations of methoxyfenozide observed in the cold season were all below the MRL. Moreover, it is necessary to monitor pesticides in the long-term to avoid their potential risks to consumers and to help the policymakers improve the set-up of MRL and PHI for crops.

## Figures and Tables

**Table 1:** Detailed information on field trials and pesticide application.

Crop Field Trials	Test Periods	Soil Types (pH/Organic Matter, %)	Dilution Rate	Number of Application	Spray Intervals (Days)	Application Solution (L/0.1 ha)	
						Methoxyfenozide	Pymetrozine
JJ-19-6	24 May–19 June	Clay loam (6.2/3.0)	2000 ^(a)^ & 5000 ^(b)^	3	5–7	151–167	159–162
YD-19-8	8–29 October	Sandy loam (7.2/3.4)			7	167–180	160–173
GH-19-9	30 October–27 November	Loam (5.9/2.5)			7	168–177	168–180

^(a)^ for methoxyfenozide ^(b)^ for pymetrozine.

**Table 2:** Optimization of MS/MS conditions for methoxyfenozide and pymetrozine.

Pesticides	Exact Mass (g/mol)	Precursor Ion (*m*/*z*)	Fragment Voltage (V)	Collision Energy (V)	Product Ions (*m*/*z*)	
					Quantification	Qualification
Methoxyfenozide	368.2	369.2	85	10/4	149.1	313.2
Pymetrozine	217.1	218.1	125	20/45	105.1	78.1

**Table 3:** The half-life and other statistical parameters for analyzed compounds in Chinese cabbage.

Test Periods	Parameters	Methoxyfenozide	Pymetrozine
May–June	Determination coefficient (R^2^)	0.62	0.76
	Dissipation rate constant (day^−1^)	0.58	0.37
	Half-life (days)	1.20	1.89
Oct.–Nov.	Determination coefficient (R^2^)	0.32	0.91
	Dissipation rate constant (day^−1^)	0.06	0.25
	Half-life (days)	11.8	2.80

## Data Availability

The data of the present study are available from the corresponding author upon request.

## Supplementary Materials

The following supporting information can be downloaded at: https://www.mdpi.com/article/10.3390/foods11192995/s1, Figure S1: Field trial locations conducted in this study; Figure S2: The chromatograms for (a)–(e) methoxyfenozide and (f)–(j) pymetrozine at 50 ng/ml standard, three levels of LOQ, and real samples analyzed by HPLC-MS/MS; Table S1: Environment conditions measured during test periods; Table S2: Detailed instrument condition of HPLC-MS/MS for methoxyfenozide and pymetrozine; Table S3: Daily cabbage consumption rate and average body weight of different gender and age.
