# Supplementary material for: Residue Behavior of Methoxyfenozide and Pymetrozine in Chinese Cabbage and Their Health Risk Assessment
Source: Foods. 2022 Sep 26;11(19):2995. doi: 10.3390/foods11192995 (PMC9563777; doi:10.3390/foods11192995)
Supplement: Supplementary file 1 [file foods-11-02995-s001.zip › foods-1905894-supplementary.pdf]

SUPPLEMENTARY MATERIALS

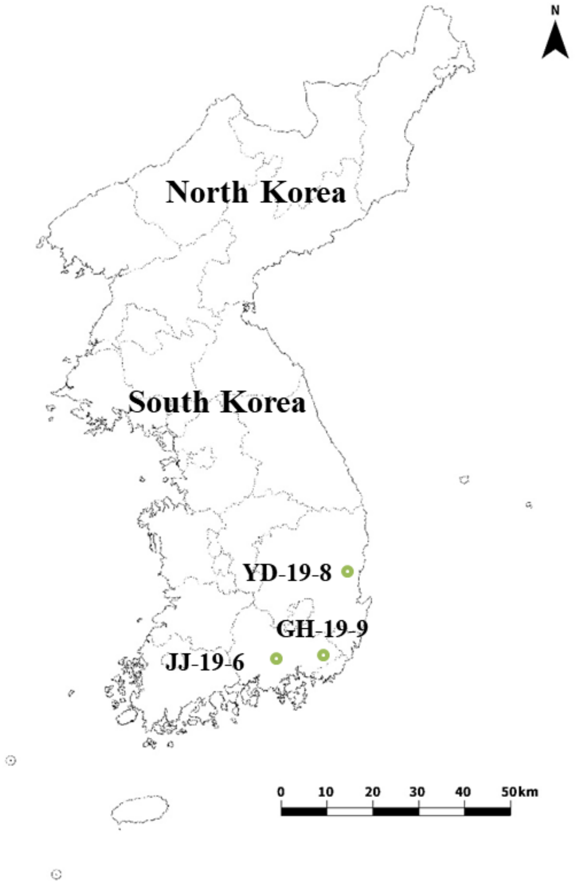

Figure S1. Field trial locations conducted in this study (NGII, 2019).

Table S1. Environment conditions measured during test periods.

Crop Field Trials	Temperature (℃)		Humidity (%)		Rainfall (mm)		Wind velocity (km/h)	
	**Min.**	**Max.**	**Min.**	**Max.**	**Min.**	**Max.**	**Min.**	**Max.**
**JJ-19-6**	9.3	32.7	50.8	89.3	0.1	66.0	2.2	6.8
	20.7 ± 1.5[a)]		71.1 ± 9.6		12.2 ± 21.6		3.9 ± 0.9	
**YD-19-8**	5.8	25.0	55.0	83.9	1.0	46.5	6.5	36.7
	15.5 ± 2.0		72.6 ± 11.0		3.8 ± 18.8		11.5 ± 6.5	
**GH-19-9**	0.0	23.8	45.3	89.0	0.5	7.0	3.6	15.1
	12.3 ± 2.9		63.5 ± 11.0		7.1 ± 5.7		6.9 ± 2.2	

[a)] Average ± Standard deviation measured during test periods

Table S2. Detailed instrument condition of HPLC-MS/MS for methoxyfenozide and pymetrozine.

Parameters	Conditions
**System**	Agilent 1260/6460 QQQMSD system
**Column**	Agilent Poroshell 120 EC-C18 (2.1 mm × 100 mm × 2.7 μm)
**Column temperature**	40℃
**Mobile phase**	0.1% formic acid in water:<br>0.1% formic acid in methanol = 33:67 (v/v)[a)], 30:70 (v/v)[b)]
**Flow rate**	0.2 mL/min
**Injection volume**	1 μL
**Ionization mode**	ESI-Positive ion mode
**Scan type**	MRM
**Gas temp.**	300℃
**Gas flow**	10 L/min

[a)] for methoxyfenozide

[b)] for pymetrozine

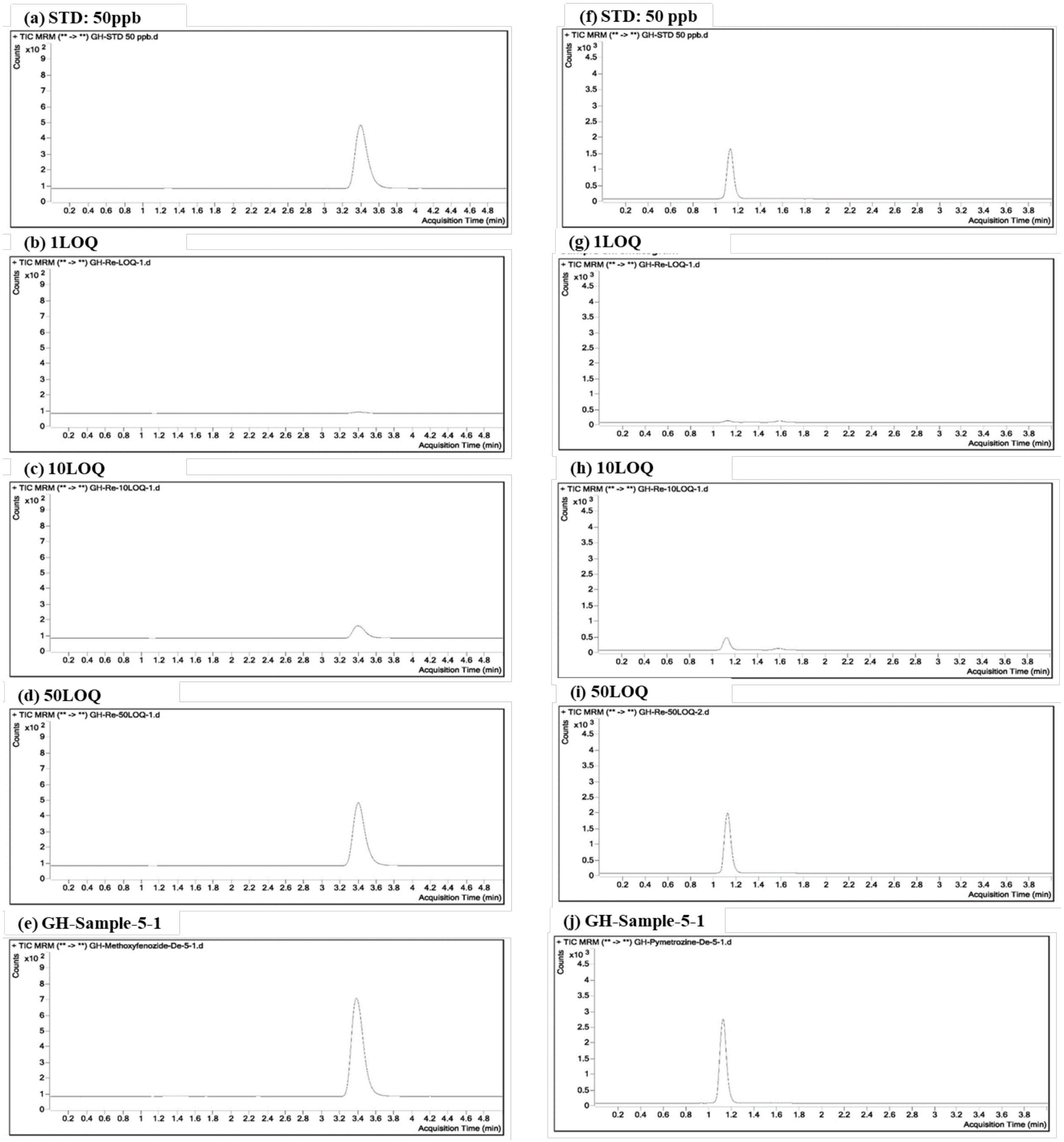

Figure S2. The chromatograms for **(a–e)** methoxyfenozide and **(f–j)** pymetrozine at 50 ng/ml standard, there levels of LOQ, and real samples analyzed by HPLC-MS/MS

Table S3. Daily cabbage consumption rate and average body weight of different gender and age.

Gender	Daily consumption rate (g/d)	Average body weight (kg)
**Male**	63.9	73.3
**Female**	42.0	58.3
